# Radiographic overestimation of inferior vena cava involvement in pediatric neuroblastoma: a case report

**DOI:** 10.1093/jscr/rjag734

**Published:** 2026-08-23

**Authors:** Serena Sha, William Cobb, Nadeen Abu Ata, Aleksander Bernshteyn

**Affiliations:** Loma Linda University School of Medicine, 11175 Campus Street, Loma Linda, CA 92350, United States; Department of General Surgery, AdventHealth Orlando, 601 E Rollins Street, Orlando, FL 32803, United States; Department of Radiology, AdventHealth Orlando, 601 E Rollins Street, Orlando, FL 32803, United States; Department of Pediatric Surgery, AdventHealth Orlando, 601 E Rollins Street, Orlando, FL 32803, United States

**Keywords:** neuroblastoma, inferior vena cava, vascular invasion, image-defined risk factors, pediatric surgical oncology, adrenal tumor

## Abstract

Neuroblastoma commonly arises from the adrenal gland and frequently demonstrates close association with major abdominal vessels. Preoperative imaging plays a critical role in surgical planning, though differentiation between true vascular invasion and extrinsic compression remains challenging. We report a case of a 2-year-old female with a right adrenal neuroblastoma in whom preoperative imaging suggested inferior vena cava involvement. Intraoperative findings demonstrated compression without invasion, allowing for safe resection without vascular reconstruction. This case highlights the limitations of cross-sectional imaging in assessing vascular invasion in neuroblastoma and underscores the importance of operative correlation when imaging findings are equivocal.

## Introduction

Neuroblastoma is the most common extracranial solid malignancy of childhood and frequently arises from the adrenal gland. Preoperative imaging plays a critical role in staging, risk stratification, and surgical planning, particularly through the identification of image-defined risk factors (IDRFs). IDRFs include tumor encasement of major vessels such as the aorta, inferior vena cava (IVC), and renal vessels, as well as organ infiltration and extension into neural foramina, and are used to predict surgical complexity and resectability.

However, neuroblastoma commonly displaces and compresses adjacent vascular structures, and distinguishing true vascular invasion from extrinsic compression can be challenging on cross-sectional imaging. Overestimation of vascular involvement may influence treatment decisions, including delay of surgical intervention and increased reliance on neoadjuvant chemotherapy. Multidisciplinary evaluation incorporating radiology, oncology, and surgical assessment is therefore essential in determining resectability of neuroblastomas, particularly given demonstrated variability in IDRF identification between radiologists and surgeons and evidence that multidisciplinary tumor board review can alter management plans [1–3].

We present a case of adrenal neuroblastoma in a young child in whom preoperative imaging suggested IVC involvement. Following multidisciplinary review, the tumor was determined to be surgically resectable, and operative findings demonstrated compression without invasion.

## Case presentation

A previously healthy, fully vaccinated 2-year-old female presented with persistent low-grade fevers (maximum temperature 101°F) and poorly localized pain involving the hip, back, and abdomen. The patient was initially evaluated at an outside emergency department for fever and limp and was diagnosed with transient synovitis. The patient’s symptoms persisted, and she presented to our institution for further workup.

On admission, vital signs were notable for tachycardia (heart rate 144 beats per minute), tachypnea (32 breaths per minute), and hypertension (blood pressure 124/104 mmHg). She was afebrile with a temperature of 98.7°F. Physical examination was significant for a palpable right-sided abdominal mass.

Laboratory evaluation demonstrated elevated inflammatory markers, with a C-reactive protein of 34 mg/l and erythrocyte sedimentation rate of 74 mm/hr. Complete blood count and comprehensive metabolic panel were otherwise unremarkable. Creatine kinase was normal, respiratory viral panel was negative, and blood and urine cultures showed no growth.

**Figure 1:**
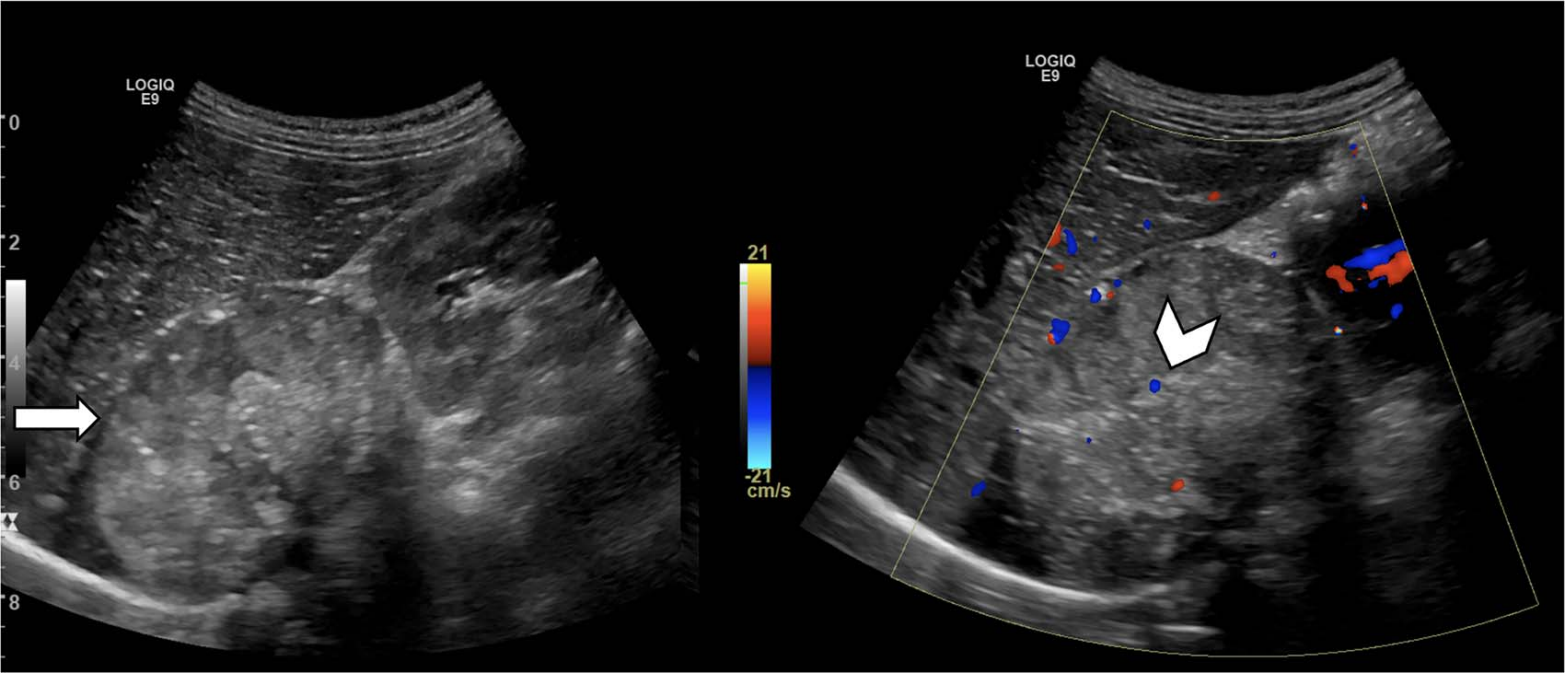
Sagittal ultrasound image demonstrates a 5 cm heterogenous right adrenal mass (arrow) with mild vascularity (arrowhead).

**Figure 2:**
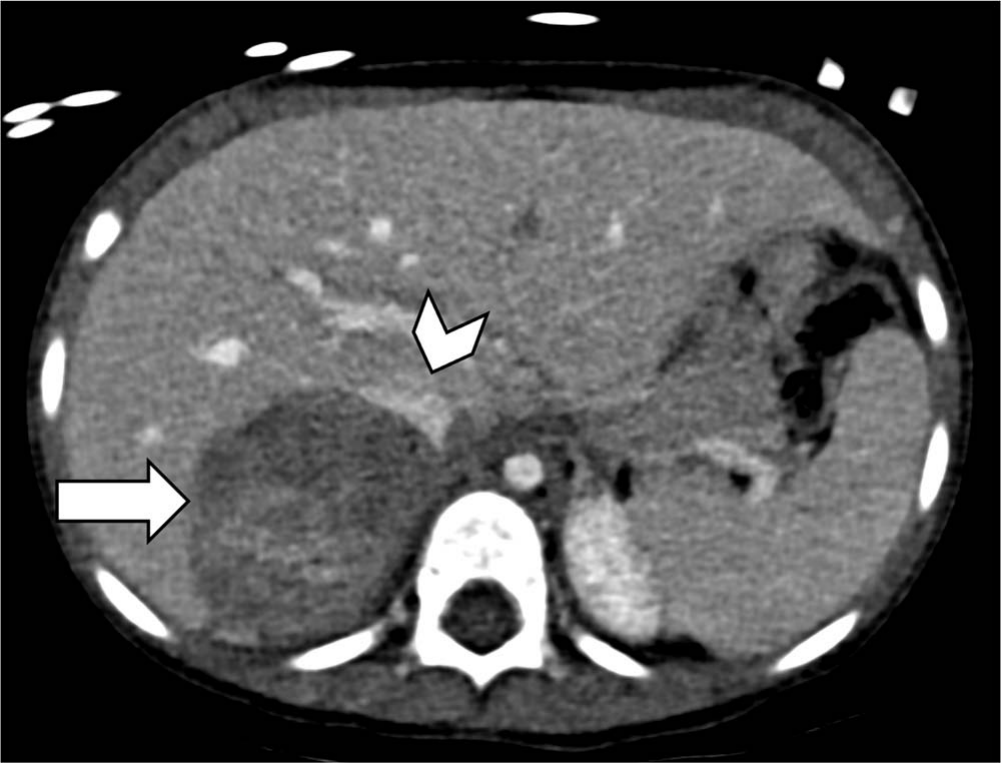
Axial CT image of the abdomen shows a 5 cm heterogenous right adrenal mass (arrow) with narrowing of the IVC that remains patent (arrowhead).

**Figure 3:**
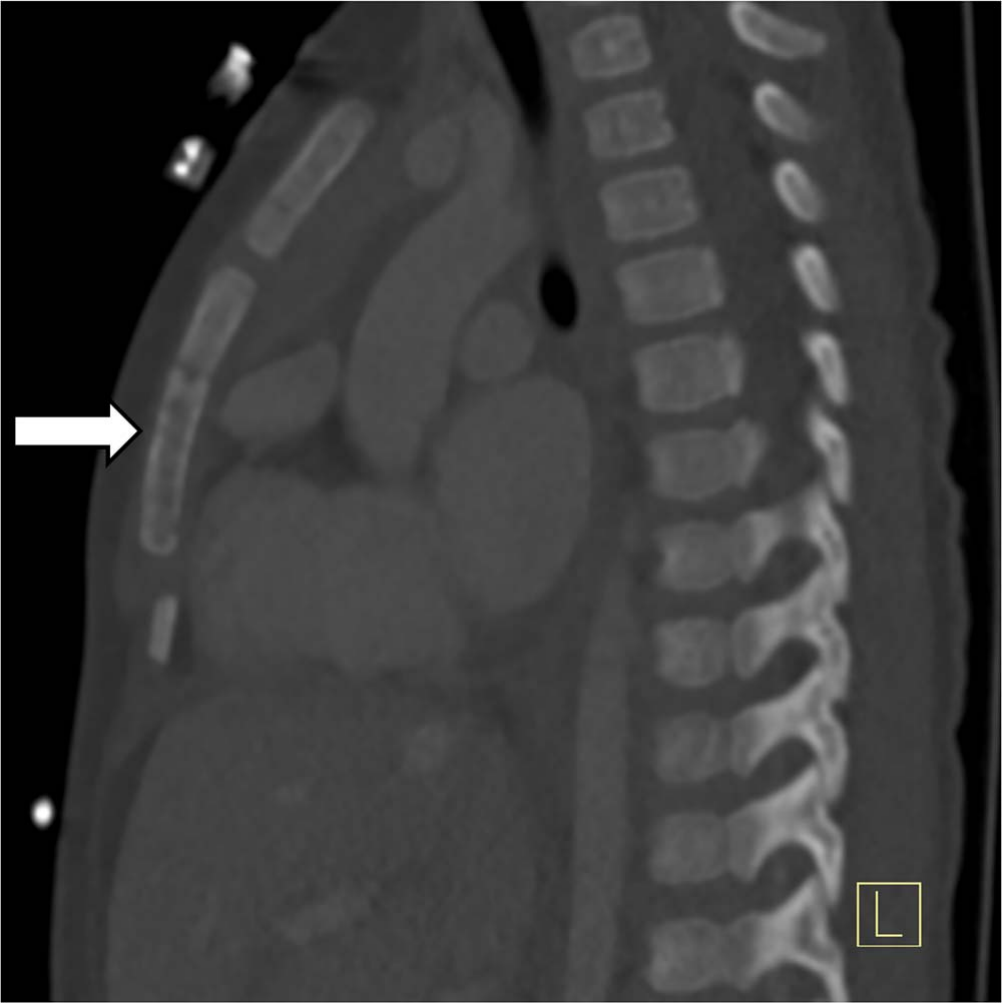
Sagittal CT image of the chest demonstrates bone marrow metastasis in the body of the sternum.

Abdominal ultrasound revealed a moderately large solid mass measuring approximately 6.0 × 5.5 × 5.5 cm, likely arising from the right adrenal gland (Fig. 1). Computed tomography (CT) of the chest, abdomen, and pelvis demonstrated a large, heterogeneous right adrenal mass most consistent with neuroblastoma. Imaging showed marked flattening of the mid intrahepatic IVC, which remained patent, raising concern for possible vascular involvement (Fig. 2). No definitive IDRFs were identified. A 1.2 × 0.4 cm right para-aortic lymph node was noted, concerning for metastatic disease. The sternum demonstrated a heterogeneous appearance suggestive of osseous involvement without discrete bone lesions (Fig. 3).

Nuclear medicine imaging demonstrated mild metaiodobenzylguanidine (MIBG) uptake in the right suprarenal mass with extensive osseous uptake, consistent with metastatic disease. Flow cytometry identified an extremely minute blast population.

Given these findings, the patient was diagnosed with suspected adrenal neuroblastoma. The multidisciplinary team elected to proceed with surgical management, including central venous access placement, adrenalectomy, and partial aortocaval lymphadenectomy.

Pathology findings showed the ‘right adrenalectomy with mass’ was neuroblastoma, poorly differentiated, 6.3 cm in greatest dimension, unfavorable histology, and the ‘aortocaval node’ was ten lymph nodes, negative for metastatic tumor (0/10).

### Operative management

The tumor was mobilized through a right subcostal incision. The IVC and renal vein were identified and preserved. Despite preoperative concern for vascular involvement, the mass was separated from the IVC without evidence of direct invasion and removed completely. Aortocaval lymphadenectomy was performed. The patient tolerated the procedure without complication.

## Discussion

Preoperative assessment of vascular involvement is critical in neuroblastoma because it influences operative planning, anticipated surgical complexity, and the use of neoadjuvant chemotherapy. The International Neuroblastoma Risk Group introduced IDRFs, including encasement of major abdominal vessels, to standardize risk stratification and predict surgical difficulty [4–6].

However, neuroblastoma frequently displaces, compresses, or encases vessels without true transmural invasion. Brisse et al. reported that tumors may demonstrate vessel flattening or circumferential contact while preserving patency, potentially leading to overestimation of invasion on imaging [7]. Although IDRF-positive tumors are associated with increased operative risk, successful resection is often possible without vascular injury [8]. Accordingly, IDRFs should be interpreted as markers of surgical risk rather than absolute unresectability.

In this case, preoperative imaging demonstrated marked flattening of the IVC with preserved patency, raising concern for vascular involvement. Intraoperatively, the tumor compressed but did not invade the IVC and was safely separated without vascular reconstruction. This imaging–operative discrepancy is consistent with prior studies demonstrating that vascular compression does not necessarily indicate true invasion [7, 8].

This case highlights the importance of multidisciplinary evaluation when vascular involvement is suspected. Imaging findings alone may overestimate invasion and influence treatment toward delayed resection or increased use of neoadjuvant therapy. In our patient, multidisciplinary review supported upfront surgery, and operative findings confirmed resectability. Careful integration of radiologic findings and surgical judgment remains essential when determining treatment strategy in neuroblastoma.

## Conclusion

This case demonstrates that apparent IVC involvement on imaging in pediatric neuroblastoma may represent extrinsic compression rather than true vascular invasion. Awareness of this diagnostic pitfall is essential for surgical planning and treatment decision-making. Operative assessment remains the gold standard for determining vascular invasion and resectability.
